# Optimal Cutoff and Accuracy of an IgM Enzyme-Linked Immunosorbent Assay for Diagnosis of Acute Scrub Typhus in Northern Thailand: an Alternative Reference Method to the IgM Immunofluorescence Assay

**DOI:** 10.1128/JCM.02744-15

**Published:** 2016-05-23

**Authors:** Stuart D. Blacksell, Cherry Lim, Ampai Tanganuchitcharnchai, Suthatip Jintaworn, Pacharee Kantipong, Allen L. Richards, Daniel H. Paris, Direk Limmathurotsakul, Nicholas P. J. Day

**Affiliations:** a Mahidol-Oxford Tropical Medicine Research Unit, Faculty of Tropical Medicine, Mahidol University, Bangkok, Thailand; b Centre for Tropical Medicine and Global Health, Nuffield Department of Clinical Medicine, University of Oxford, Oxford, United Kingdom; c Chiangrai Prachanukhru Hospital, Chiangrai, Thailand; d Viral and Rickettsial Diseases Department, Naval Medical Research Center, Silver Spring, Maryland, USA; e Department of Preventive Medicine and Biostatistics, Uniformed Services University of the Health Sciences, Bethesda, Maryland, USA; f Department of Tropical Hygiene, Faculty of Tropical Medicine, Mahidol University, Bangkok, Thailand; Memorial Sloan-Kettering Cancer Center

## Abstract

The enzyme-linked immunosorbent assay (ELISA) has been proposed as an alternative serologic diagnostic test to the indirect immunofluorescence assay (IFA) for scrub typhus. Here, we systematically determine the optimal sample dilution and cutoff optical density (OD) and estimate the accuracy of IgM ELISA using Bayesian latent class models (LCMs). Data from 135 patients with undifferentiated fever were reevaluated using Bayesian LCMs. Every patient was evaluated for the presence of an eschar and tested with a blood culture for Orientia tsutsugamushi, three different PCR assays, and an IgM IFA. The IgM ELISA was performed for every sample at sample dilutions from 1:100 to 1:102,400 using crude whole-cell antigens of the Karp, Kato, and Gilliam strains of O. tsutsugamushi developed by the Naval Medical Research Center. We used Bayesian LCMs to generate unbiased receiver operating characteristic curves and found that the sample dilution of 1:400 was optimal for the IgM ELISA. With the optimal cutoff OD of 1.474 at a sample dilution of 1:400, the IgM ELISA had a sensitivity of 85.7% (95% credible interval [CrI], 77.4% to 86.7%) and a specificity of 98.1% (95% CrI, 97.2% to 100%) using paired samples. For the ELISA, the OD could be determined objectively and quickly, in contrast to the reading of IFA slides, which was both subjective and labor-intensive. The IgM ELISA for scrub typhus has high diagnostic accuracy and is less subjective than the IgM IFA. We suggest that the IgM ELISA may be used as an alternative reference test to the IgM IFA for the serological diagnosis of scrub typhus.

## INTRODUCTION

Scrub typhus and murine typhus are important causes of acute febrile illness in Thailand and Laos (1–6). Diagnosis of scrub typhus still relies on serology due to the convenience of sample collection; the relative simplicity of the assays; and the expense, complexities, and deficiencies in sensitivity of other modalities (5, 7, 8). The indirect immunofluorescence assay (IFA) is the serological reference standard despite being inconsistent in terms of antigen composition, diagnostic cutoff (9), and repeatability (10). Enzyme-linked immunosorbent assays (ELISAs) offer a number of advantages over IFA, particularly in terms of simplicity, reading objectivity, and sample throughput. Use of ELISA for acute scrub typhus diagnosis has been previously evaluated (11, 12); however, its optimal sample dilution and cutoff optical density (OD) have not been determined.

Defining the cutoff value of a new serological test is conventionally done by comparing the results of the serological test of interest with the results of a reference test. However, this conventional approach assumes that the reference test has perfect sensitivity and specificity; if this is not the case, then estimates of sensitivity and specificity for the test under evaluation will be biased and the selected cutoff value suboptimal (13). Bayesian latent class models (LCMs) are increasingly used to determine the optimal cutoff value and to estimate the accuracy of a diagnostic test, since they do not need to assume that the accuracy of reference tests is perfect (14–17). We recently used Bayesian LCMs to reanalyze our existing data set from a previously published prospective study (18) and showed that an IgM IFA with a cutoff titer of ≥1:12,800 in admission samples or a 4-fold rise to ≥1:200 in convalescent/discharge samples had low sensitivity and specificity (70.0% and 83.8%, respectively) (19) and that optimal cutoff titers for IgM IFA of ≥1:3,200 in admission specimens or a ≥4-fold rise to ≥1:3,200 in convalescent/discharge specimens provided higher sensitivity and specificity (81.6% and 100%, respectively) (20).

In this study, we used Bayesian LCMs and blood samples collected from our previously published prospective study (18) to systematically determine the optimal sample dilution and optimal cutoff OD and to examine the accuracy of the Naval Medical Research Center (NMRC) “in-house” scrub typhus IgM ELISA.

## MATERIALS AND METHODS

### Study patients and blood samples.

The data set and blood samples used in this study were from patients recruited into a prospective study of acute febrile illness from August 2007 to August 2008 in Chiangrai, Thailand (18). In brief, 161 patients over 15 years old presenting with acute fever of less than 2 weeks, with three negative malaria blood smears and no evidence of a primary focus of infection, were recruited into the study (18). Every patient was examined for the presence of an eschar. Admission blood samples were collected and tested using *in vitro* culture for Orientia tsutsugamushi, a nested-PCR (nPCR) assay targeting the 56-kDa gene, a 47-kDa gene-based quantitative real-time PCR (qPCR) assay, a *groEL*-based qPCR assay, and an IgM IFA. Convalescent-phase or hospital discharge (convalescent/discharge) blood samples were also collected and tested by IgM IFA. All the blood samples were kept at −80**°**C and tested using the IgM ELISA in 2014.

### Ethical statement.

Ethical approval for the prospective study was obtained from the ethical committee of Chiangrai Hospital; the Ministry of Public Health, Thailand; and the Oxford Tropical Research Ethics Committee, United Kingdom. Signed written informed consent was obtained from every patient before sample collection (18).

### Diagnostic tests.

*In vitro* isolation of O. tsutsugamushi (culture) (21) and the 56-kDa gene nPCR assay (22), 47-kDa-based qPCR assay (23), and *groEL*-based qPCR assay (24) were performed as described previously. The IgM IFA was performed using pooled Karp, Kato, and Gilliam reference strain O. tsutsugamushi antigens as described previously (18). In short, IgM antibodies were detected using IFA slides produced by the Australian Rickettsial Reference Laboratory (ARRL) (Geelong, Australia). Patient sera were serially 2-fold diluted from 1:100 to 1:25,600, and the endpoint was determined as the highest titer displaying specific fluorescence (18).

The IgM ELISA was essentially the same as that previously described by Suwanabun et al. (11). All ELISAs were performed at the Mahidol-Oxford Tropical Medicine Research Unit (MORU), Bangkok, Thailand. In brief, whole-cell antigen lysates of Karp, Kato, and Gilliam reference strains of O. tsutsugamushi and mock-infected cell lysate produced at the Viral and Rickettsial Diseases Department of the NMRC, Silver Spring, Maryland, USA (11, 12), were used as ELISA antigens. The scrub typhus antigen quality was assessed for each strain of O. tsutsugamushi Karp, Kato, and Gilliam for identity (qPCR and sequencing) and adventitious contamination (blind culture to blood agar plates, thioglycolate broth, and qPCR for mycoplasma). To standardize the antigen preparations, each strain was assessed for reactivity with reference scrub typhus-positive and -negative human serum controls at the NMRC. Following assessment, the reactivity of each antigen strain was compared to those of previous antigen batches, and the concentration was adjusted to permit the use of the component antigens at a standard dilution of 1:1,000 in coating buffer. In this evaluation, a single batch of component antigens was used at the MORU. All ELISAs were performed by two experienced operators (A.T. and S.J.). Serum samples were serially 2-fold diluted from 1:100 to 1:102,400. Negative- and positive-control samples were used as a control for within- and between-day consistency and were included in four wells each on each plate (2 per control sample). Bound anti-O. tsutsugamushi IgM antibodies were detected with anti-human IgM peroxidase conjugate (Invitrogen Corporation, USA) and tetramethylbenzidine substrate (KPL Inc., Maryland, USA). The OD was read at a wavelength of 450 nm (minus a reference OD value read at 650 nm) with a microtiter plate reader (Multiskan FC; Thermo Scientific, Finland). The ODs from the mock-antigen wells were subtracted as background absorbance to give a final average total net absorbance (net OD, or OD at 450 nm).

### Statistical analysis.

The objective of the study was to determine an optimal sample dilution and a single optimal cutoff titer for IgM ELISA that provided the highest accuracy, not only when an admission sample was initially available, but also when a convalescent/discharge sample was available. Therefore, only patients with both admission and convalescent/discharge samples were included in the study, and we performed two stages of statistical analysis.

The first stage represented the acute clinical situation where only an admission sample was available. We used Bayesian LCMs to generate unbiased receiver operating characteristic (ROC) curves for the sensitivities and specificities of all possible cutoff titers of IgM ELISA in the admission sample alone, without using the convalescent/discharge sample IgM IFA and IgM ELISA results. We performed this for all possible sample dilutions. In brief, Bayesian LCMs estimated prevalence and the sensitivity and specificity of each diagnostic test with their 95% credible intervals (CrIs) using the Markov Chain Monte Carlo (MCMC) method (17). Bayesian LCMs do not assume that any diagnostic test or combination of diagnostic tests is perfect. The true disease status of each patient was estimated by the model in each MCMC iteration and expressed as the overall disease prevalence. The diagnostic tests included in the model were culture, a combination of PCR assays, IgM IFA for the admission sample alone, ELISA IgM for the admission sample alone, and presence of an eschar. The combination of PCR assays was considered positive when at least two out of three PCR assays, targeting the 56-kDa, 47-kDa, and *groEL* genes, were positive, as previously described (18). Models that took account of correlation between the IgM IFA and the IgM ELISA were used (19, 20, 25). The IgM IFA in the first model was considered positive when the admission IgM IFA titer was ≥1:3,200 (20). Unbiased ROC curves were generated as previously described (20).

The second stage of analysis represented the situation when a convalescent/discharge sample was available. We used Bayesian LCMs to generate unbiased ROC curves for the sensitivities and specificities of all possible cutoff titers of IgM ELISA using paired samples and for all possible sample dilutions. The higher OD of either the admission sample or the convalescent/discharge sample was used. The IgM IFA in the second model was considered positive when the admission IgM IFA titer was ≥1:3,200 or there was a 4-fold rise to ≥1:3,200 in convalescent/discharge samples compared to admission samples (20).

The optimal sample dilution was selected by considering that it should provide a cutoff OD between 1.00 and 2.00, which was recommended by the microtiter plate reader manufacturer as the central and optimal regions of the linear detection range (0 to 3 OD units) (Thermoscientific). The optimal cutoff OD was selected by considering that it should provide the highest accuracy in both situations (i.e., an admission sample alone and with paired convalescent/discharge samples). We evaluated the sensitivity, specificity, and accuracy of all possible ODs between the recommended cutoff ODs suggested by both models side by side. The OD that had the highest overall accuracy was selected as the optimal cutoff OD.

All Bayesian LCMs assumed that no prior information (noninformative priors) about the unknown parameters (i.e., prevalence, sensitivities, and specificities) was available, except that the specificity of the culture was fixed at 100%. Bayesian LCMs were performed in WinBUGS 1.4 (26). Texts S1 and S2 in the supplemental material provide full data sets and the models used, respectively.

### *Post hoc* model evaluation.

To validate the sensitivity of the IgM ELISA estimated by the Bayesian LCMs, we estimated the naive sensitivity of the IgM ELISA in the patients who had a firm diagnosis of scrub typhus. The firm diagnosis of scrub typhus was made on the basis of either a positive blood culture, a combination of positive PCR assays, or the presence of an eschar in our data set. To validate the specificity of the IgM ELISA estimated by the Bayesian LCMs, we estimated the naive specificity of the IgM ELISA results in patients with a firm single diagnosis of other diseases, including murine typhus and dengue, in the data set described previously (19, 20). The diagnosis of murine typhus was made if there was a ≥4-fold increase in the Rickettsia typhi IgM IFA titer between paired samples (using slides coated with R. typhi strain Wilmington; ARRL, Geelong, Australia) (19, 20). The diagnosis of dengue was defined by the detection of NS1 antigen using Panbio ELISAs and IgM antibodies (Panbio, Brisbane, Australia) in paired samples (19, 20).

### Evaluation of ELISA repeatability.

Between- and within-day variation was determined by calculating the mean and the standard deviation (SD) of the positive control on each plate to derive the between-plate, between-day, and within-day percent coefficient of variation (%CV) as follows: %CV = (SD/mean) × 100. Median values and interquartile ranges (IQR) were also calculated for the population.

## RESULTS

**FIG 1:**
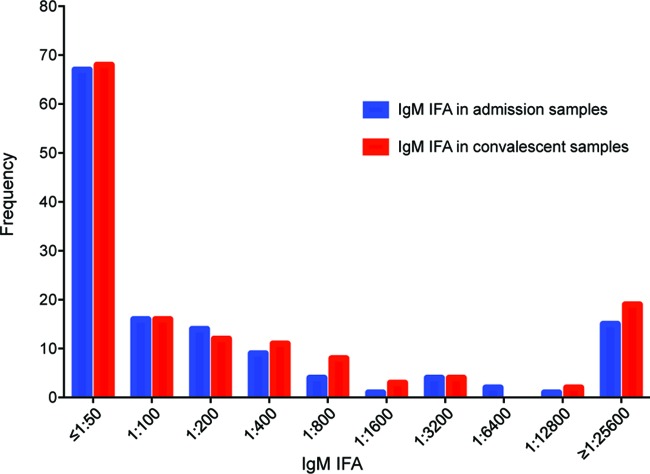
Distribution of IgM IFA titers in admission samples and convalescent/discharge samples.

Of 161 patients with acute undifferentiated fever evaluated, 135 (83.8%) had both acute and convalescent/discharge serum samples available for ELISA IgM and were included in the study. The median age was 42 years (IQR, 29 to 52 years; range, 15 to 84 years), and 83/135 (63%) were male. The median duration of fever before admission was 5 days (IQR, 4 to 7 days). The median duration between the admission and convalescent/discharge sample was 12 days (IQR, 3 to 14 days). Culture, a combination of PCR assays, IgM IFA, and the presence of an eschar were positive in 7, 22, 25, and 14 patients, respectively. There was a dominance of IFA titers at the lower end of the dilution scale for IgM titers in both admission and convalescent/discharge samples (Fig. 1).

**FIG 2:**
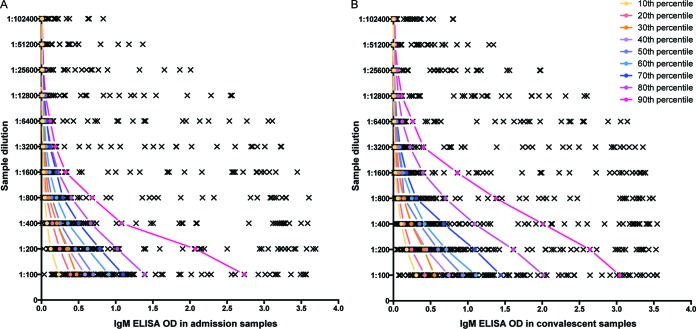
Distribution of IgM ELISA ODs in admission samples (A) and convalescent/discharge samples (B) at sample dilutions from 1:100 to 1:102,400. The colored lines show the positions of the patients who had the OD at the 10th, 20th, and every 10th percentile up to the 90th percentile at a sample dilution of 1:100.

The distribution of IgM ELISA ODs was skewed to the right for all sample dilutions and for both admission and convalescent/discharge samples (Fig. 2A and B). The IgM ELISA ODs in convalescent/discharge samples were generally higher than those in admission samples for all sample dilutions (all *P* values < 0.001; Wilcoxon paired signed-rank test). For example, at a sample dilution of 1:100, 86 (63.7%) patients had ODs in the convalescent/discharge sample higher than that in the admission sample. Patients who had a high OD at a 1:100 sample dilution also had comparatively high ODs at every dilution for both admission and convalescent/discharge samples. For example, Fig. 2A shows that the patient who had an IgM ELISA OD in the 10th percentile in the admission sample at a 1:100 sample dilution always had IgM ELISA ODs higher than those of the patients who had IgM ELISA ODs in the 20th, and 30th to 90th percentiles in all sample dilutions. The OD difference between each 10th percentile was narrower at the higher sample dilution (Fig. 2A and B).

Using the ROC curve to evaluate the performance of IgM ELISA with the admission sample alone, we found that the recommended cutoff ODs ranged from 1.854 to 0.028, decreasing with increasing dilution (see Fig. S1 in the supplemental material). For paired samples, the recommended cutoff ODs ranged from 2.427 to 0.053 for the dilution range 1:100 to 1:102,400 (see Fig. S2 in the supplemental material). A dilution of 1:400 was selected as the recommended sample dilution because it had recommended cutoff OD values for both scenarios between 1.00 and 2.00 (see Fig. S1 and S2 in the supplemental material), i.e., in the middle of the linear range of the microtiter plate reader.

To select a single optimal cutoff OD at a sample dilution of 1:400, the sensitivities and specificities of all possible ODs were evaluated between the recommended OD range when only the admission sample was available (OD = 1.010) and that when the paired convalescent/discharge samples were available (OD = 1.474) (Table 1). An OD of 1.474 was selected as the optimal cutoff because it provided the highest overall accuracy. Using a cutoff OD of 1.474 at a sample dilution of 1:400, Bayesian LCMs estimated that the IgM ELISA had a sensitivity of 69.0% (95% CrI, 60.6% to 76.9%) and a specificity of 100% (95% CrI, 99.1% to 100%) for the admission sample alone and a sensitivity of 85.7% (95% CrI, 77.4 to 86.7%) and a specificity of 98.1% (95% CrI, 97.2 to 100%) with the addition of the paired samples (Table 2). The Bayesian LCM estimated that there were 29 scrub typhus patients (95% CrI, 27 to 33 patients), representing a prevalence of scrub typhus at 21.8% (95% CrI, 15.1% to 29.5%) in the study participants. There was no significant difference between the sensitivities of the IgM ELISA and the IgM IFA for admission samples alone (69.0% versus 69.0%; Bayesian *P* value = 0.50) or between the sensitivities of the two tests using paired samples (85.7% versus 86.2%; Bayesian *P* value = 0.31).

**TABLE 1:** Sensitivity and specificity of IgM ELISA at a sample dilution of 1:400

Cutoff OD	Model using^*a*^:				Overall accuracy (%)^*b*^
	On-admission sample alone		Paired samples		
	Sensitivity	Specificity	Sensitivity	Specificity	
1.010	78.6 (68.8–85.2)	100 (98.2–100)	85.2 (77.4–87.1)	93.5 (92.5–96.2)	93.3
1.023	73.3 (64.7–81.5)	100 (98.2–100)	85.2 (77.4–87.1)	93.5 (92.5–96.2)	93.0
1.080	73.3 (64.7–81.5)	100 (98.2–100)	85.2 (77.4–87.1)	94.4 (93.5–97.1)	93.3
1.093	70.0 (61.3–77.8)	100 (98.2–100)	85.2 (77.4–87.1)	94.4 (93.5–97.1)	93.0
1.165	70.0 (61.3–77.8)	100 (98.2–100)	85.2 (77.4–87.1)	95.3 (94.4–98.1)	93.3
1.214	70.0 (61.3–77.8)	100 (98.2–100)	85.2 (77.4–87.1)	95.3 (94.4–98.1)	93.3
1.273	70.0 (61.3–77.8)	100 (98.2–100)	85.2 (77.4–87.1)	96.3 (95.3–99.0)	93.7
1.275	70.0 (61.3–77.8)	100 (98.2–100)	85.2 (77.4–87.1)	96.3 (95.3–99.0)	93.7
1.307	69.0 (60.6–76.9)	100 (99.1–100)	85.2 (77.4–87.1)	97.2 (96.3–100)	94.1
1.474	69.0 (60.6–76.9)	100 (99.1–100)	85.7 (77.4–86.7)	98.1 (97.2–100)	94.4

a The values are medians and 95% CrIs.

b Overall accuracy is an average of the accuracies estimated by both models.

**TABLE 2:** Accuracies of diagnostic tests for acute scrub typhus estimated using Bayesian LCMs

Parameter^*a*^	Accuracy (%) for model using^*b*^:	
	On-admission sample alone	Paired samples
*In vitro* culture for O. tsutsugamushi		
Sensitivity	24.1 (21.2–26.9)	24.1 (21.2–25.9)
Specificity	100	100
PPV	100	100
NPV	82.8 (79.7–85.2)	82.8 (79.7–84.4)
Combination of PCR assays^*c*^		
Sensitivity	69.2 (63.3–73.3)	66.7 (61.3–70.0)
Specificity	98.1 (95.5–100)	97.2 (96.3–99.1)
PPV	90.9 (77.3–100)	86.4 (81.8–95.5)
NPV	92.0 (89.4–92.9)	91.2 (89.4–92.0)
IgM IFA^*d*^		
Sensitivity	69.0 (60.6–76.9)	86.2 (75.8–92.6)
Specificity	100 (99.1–100)	100 (99.1–100)
PPV	100 (95.0–100)	100 (96.0–100)
NPV	92.2 (88.7–94.8)	96.4 (92.7–98.2)
IgM ELISA^*e*^		
Sensitivity	69.0 (60.6–76.9)	85.7 (77.4–86.7)
Specificity	100 (99.1–100)	98.1 (97.2–100)
PPV	100 (95.0–100)	92.3 (88.5–100)
NPV	92.2 (88.7–94.8)	96.3 (93.6–96.3)
Presence of eschar		
Sensitivity	42.9 (37.9–46.7)	41.4 (37.5–44.8)
Specificity	98.1 (97.2–100)	98.1 (98.1–99.1)
PPV	85.7 (78.6–100)	85.7 (85.7–92.9)
NPV	86.8 (83.5–87.6)	86.0 (83.5–87.6)

a PPV positive predictive value; NPV negative predictive value.

b The values are medians and 95% CrIs.

c A combination of PCR assays was defined as positive when at least two out of the three PCR assays (56-kDa nPCR assay, 47-kDa-based qPCR assay, and *groEL*-based qPCR assay) were positive.

d In the model using on-admission samples alone, IgM IFA was defined as positive in those with an admission IgM IFA titer of ≥1:3,200. In the model using paired samples, IgM IFA was defined as positive in those with an admission IgM IFA titer of ≥1:3,200 or at least a 4-fold rise to ≥1:3,200 in the convalescent/discharge IgM IFA titer compared to the admission IgM IFA titer.

e In the model using on-admission samples alone, IgM ELISA was defined as positive when the OD was ≥1.474 at a sample dilution of 1:400. In the model using paired samples, IgM ELISA was defined as positive when the OD was ≥1.474 at a sample dilution of 1:400 in either sample.

### *Post hoc* model evaluation.

We evaluated the robustness of the cutoff OD of 1.474 at a sample dilution of 1:400 and showed that the IgM ELISA had a naive sensitivity of 69.0% (20/29) using paired samples in patients with a firm diagnosis of scrub typhus made using other diagnostic tests (Table 3). Eight of the nine patients with negative IgM ELISA results had very low IgM ELISA ODs (<0.5), even though the overall durations between the onset of fever and the convalescent/discharge sample date were 5 to 10 days (*n* = 3) and more than 10 days (*n* = 5) (see Table S1 in the supplemental material). The naive specificity of IgM ELISA in patients with a firm single diagnosis of either murine typhus or dengue virus infection was 96.3% (26/27), using paired samples in this group of patients (Table 3).

**TABLE 3:** Naive sensitivity of IgM ELISA estimated in those in the study cohort who had a positive blood culture, a combination of PCR assays positive, or the presence of an eschar and in those who had a final diagnosis of either murine typhus or dengue infection

Population	No. with IgM ELISA^*a*^ positive	Naive sensitivity (%)	No. with IgM ELISA^*a*^ negative	Naive specificity (%)
Patients who had blood culture positive (*n* = 7)	5	71.4 (5/7)	NA	NA
Patients who had a combination of PCR assays positive^*b*^ (*n* = 22)	17	77.3 (17/22)	NA	NA
Patients with the presence of an eschar (*n* = 14)	11	78.6 (11/14)	NA	NA
Overall^*c*^ (*n* = 29)	20	69.0 (20/29)	NA	NA
Patients with a final diagnosis of murine typhus (*n* = 8)	NA^*d*^	NA	8	100 (8/8)
Patients with a final diagnosis of dengue (*n* = 19)	NA	NA	18	94.7 (18/19)
Overall (*n* = 27)	NA	NA	26	96.3 (26/27)

a IgM ELISA was defined as positive when the OD was ≥1.474 at a sample dilution of 1:400 for either the admission or the convalescent/discharge sample.

b A combination of PCR assays was defined as positive when at least two out of the three PCR assays (56-kDa nPCR assay, 47-kDa-based qPCR assay, and *groEL*-based qPCR assay) were positive.

c Patients who had blood culture positive, a combination of PCR assays positive, or the presence of an eschar.

d NA, not applicable.

### ELISA repeatability.

One hundred and fifty-five ELISA plates were processed on 23 separate days. The minimum number of ELISA plates processed on a single day was 2 plates (days 1 and 2), and the maximum was 10 plates (days 12 and 21) (median, 8 plates/day). Between-plate positive-control values (*n* = 310 observations) gave a median OD of 2.99 with a %CV of 9.24% (mean, 2.92%; SD, 0.27%; IQR, 2.79% to 3.09%). Between-day mean positive-control values (*n* = 23 observations) gave a median OD of 3.02 and a %CV of 7.43% (mean, 2.96%; SD, 0.22%; IQR, 2.61% to 3.09%). Within-day variation for the positive-control values expressed as %CV gave a median of 4.0% (mean, 3.69%; SD, 1.81%; IQR, 3.0% to 4.0%).

## DISCUSSION

We have described an unbiased approach using Bayesian LCMs to define optimal sample dilution and optimal cutoff OD values for the use of an IgM ELISA for the diagnosis of acute scrub typhus. We demonstrated that the sensitivity and specificity of the IgM ELISA are comparable to those of the IgM IFA with a cutoff of ≥1:3,200 in admission samples or a 4-fold rise to ≥1:3,200 in convalescent/discharge samples compared to admission samples and suggest that the IgM ELISA could be used as an alternative serological reference test instead of the IgM IFA.

In addition to comparable accuracy, there are a number of other reasons why ELISA technologies could replace IFA as the scrub typhus reference serological test. In general, the IFA is a subjective semiquantitative test that requires highly trained operators to prepare the slides and determine the results (9, 10). Furthermore, the IFA does not lend itself to the testing of large numbers of samples, hindering throughput when performing clinical studies or in clinical settings with large numbers of patients. These problems could easily be overcome by the ELISA, which could be automated and is easier to standardize, as it provides fully quantitative and consistent results (11, 12).

The sensitivity and specificity values estimated by our study are unbiased, because they are based on the true status of patients predicted by the Bayesian LCM. Previous studies reported that the NMRC IgM ELISA had higher sensitivity and lower specificity (11, 12) than the immunoperoxidase (IIP) assay (the IIP assay is analogous to IFA with the exception that different substrates are employed) titer of 1:400, which was assumed to be “perfect.” Suwanabun et al. reported that the NMRC IgM ELISA had a sensitivity of 92.9% and a specificity of 93.6% (11). Coleman et al. reported that the NMRC IgM ELISA at a sample dilution of 1:400 had a sensitivity of 94.2% and a specificity of 91.3% (12). However, the sensitivities reported by Suwannabun and Coleman were predicted to be overestimated, and this could be because low cutoff ODs were used and sensitivity was biased toward a group of patients who had IIP assay titers of ≥1:400. The specificities reported by Suwanabun and Coleman could be underestimated because low cutoff ODs were used and the IgM ELISA could be positive in scrub typhus patients who had an IIP assay titer of <1:400. These problems were due to disease misclassification by the imperfect gold standard (14–17). In contrast, the sensitivity and specificity values estimated by our study were not biased toward an imperfect reference test. This is also supported by the *post hoc* model evaluation showing that the sensitivity and specificity estimated by the Bayesian LCMs are comparable to the naive sensitivity and specificity values estimated in a group of patients with firm diagnoses of scrub typhus and of other diseases, respectively.

The choice of an appropriate sample dilution and diagnostic cutoff is an important step in diagnostic test validation and one that is often overlooked. Often, a generic sample dilution and a cutoff are defined based on comparing the results with the results of the reference tests, which are rarely if ever perfect for diagnosing tropical infectious diseases. Suwanabun et al. used a healthy control group to define the cutoff for the IgM ELISA (11), while Coleman et al. used an IIP assay titer of 1:400 to define the cutoff for the ELISA (12). The rationale for the sample dilution selection was unclear (11, 12). Bayesian LCMs and the generation of unbiased ROC curves have been increasingly used for many diseases (14, 16, 27, 28). In this study, we also show that the selection of sample dilution for the IgM ELISA could be systematically performed. Although all sample dilutions were capable of providing comparable accuracies using the IgM ELISA if an optimal cutoff OD was chosen (see Fig. S1 and S2 in the supplemental material), an optimal sample dilution can be recommended that fits with the optimal OD reading range recommended by the ELISA reader manufacturers. Application of the methodology described here would lead to a broader understanding of the utility of reference tests in the evaluation of quantitative diagnostic tests. This could lead to changes in the diagnostic process and clinical practice for many infectious diseases.

This study has some limitations. First, the sample size in our study was small. Second, the antigenic variation characteristic of O. tsutsugamushi strains can affect assay sensitivity and specificity. Previous studies have characterized the O. tsutsugamushi strains causing human disease (29–34) and presenting in vectors (35–37) in Thailand, showing a dominance of Karp- and Gilliam-like genotype strains, as well as TA716 and TA763 genotype strains. However, the O. tsutsugamushi strains that cause disease in the Chiangrai locality have not been fully characterized, and the incorporation of contemporary strains into the antigenic mix used in the test may increase the sensitivity. This is supported by our finding that some patients who had a firm diagnosis of scrub typhus were IgM ELISA negative, despite the time between the onset of fever and convalescent/discharge sampling being more than 10 days (see Table S1 in the supplemental material). An IgM ELISA using well-characterized contemporary local strain recombinant O. tsutsugamushi 56-kDa outer membrane antigens (38, 39) could be developed and evaluated in clinical settings using appropriate statistical models. Third, the accuracy of diagnostic tests varies based on prevalence, clinical variability, and availability and timing of convalescent-phase samples (40). Further studies to evaluate the optimal cutoff titers and accuracy of the IgM ELISA in different settings are still required.

In conclusion, we propose that IgM ELISA should be used as an alternative serological reference test for acute scrub typhus in the locality of Chiangrai, Thailand, with the recommendation that geographically specific diagnostic cutoffs be determined for other localities and employed using appropriate statistical models.

## Supplementary Material

Supplemental material
