# Supplementary material for: Optimal Cutoff and Accuracy of an IgM Enzyme-Linked Immunosorbent Assay for Diagnosis of Acute Scrub Typhus in Northern Thailand: an Alternative Reference Method to the IgM Immunofluorescence Assay
Source: J Clin Microbiol. 2016 May 23;54(6):1472–8. doi: 10.1128/JCM.02744-15 (PMC4879268; doi:10.1128/JCM.02744-15)
Supplement: Supplemental material [file supp_54_6_1472__index.html]

Optimal Cutoff and Accuracy of an IgM Enzyme-Linked Immunosorbent Assay for Diagnosis of Acute Scrub Typhus in Northern Thailand: an Alternative Reference Method to the IgM Immunofluorescence Assay — Supplemental material 

# Optimal Cutoff and Accuracy of an IgM Enzyme-Linked Immunosorbent Assay for Diagnosis of Acute Scrub Typhus in Northern Thailand: an Alternative Reference Method to the IgM Immunofluorescence Assay

## Supplemental material

- Supplemental file 1 -

  Table S1 (IgM ELISA OD for admission-, convalescent-, and discharge-phase samples at a 1:400 sample dilution for 29 patients with blood culture positive for *O. tsutsugamushi*, positive results for a combination of PCR assays, or the presence of an eschar)

  PDF, 83K
- Supplemental file 2 -

  Fig. S1 (Unbiased ROC curves obtained using Bayesian LCMs for all possible cutoff ODs for IgM ELISA at sample dilutions from 1:100 to 1:102,400 for admission-phase samples alone)

  PDF, 403K
- Supplemental file 3 -

  Fig. S2 (Unbiased ROC curves obtained using Bayesian LCMs for all possible cutoff ODs for IgM ELISA at sample dilutions from 1:100 to 1:102,400 for paired convalescent-phase samples)

  PDF, 415K
- Supplemental file 4 -

  Text S1 (Data set for Bayesian latent class models)

  PDF, 100K
- Supplemental file 5 -

  Text S2 (WinBUGS models)

  PDF, 77K
