# Supplementary material for: Optimal Cutoff and Accuracy of an IgM Enzyme-Linked Immunosorbent Assay for Diagnosis of Acute Scrub Typhus in Northern Thailand: an Alternative Reference Method to the IgM Immunofluorescence Assay
Source: J Clin Microbiol. 2016 May 23;54(6):1472–8. doi: 10.1128/JCM.02744-15 (PMC4879268; doi:10.1128/JCM.02744-15)
Supplement: Supplemental material [file JCM.02744-15_zjm999094981so1.pdf]

Supplementary Table 1. IgM ELISA OD in admission sample and convalescent/discharge sample at sample dilution 1:400 in 29 patients who had either blood culture positive for *O. tsutsugamushi*, a combination of PCR assays positive or the presence of eschar

Gender and age (years)	Duration of fever prior to admission (days)	Duration between the admission sample and convalescent sample (days)	IgM ELISA OD in the admission sample[a]	IgM ELISA OD in the convalescent sample[a]	Culture	PCR assays[b]	Presence of an eschar
**M 19**	14	6	**0.048**	**0.070**	-	+	+
**M 48**	5	12	3.237	3.176	-	-	+
**F 51**	9	3	1.474	**1.165**	-	-	+
**M 52**	10	3	3.285	3.211	-	-	+
**F 40**	8	26	3.177	3.25	+	+	+
**M 28**	2	4	**1.08**	1.677	-	+	-
**M 42**	NA	14	**0.071**	**0.086**	-	+	-
**M 84**	3	10	**0.056**	**0.09**	+	-	-
**M 71**	3	8	**0.082**	**0.107**	-	+	-
**F 34**	5	46	3.118	3.229	+	+	+
**F 38**	7	1	2.095	1.917	-	+	-
**F 65**	7	2	**0.058**	**0.041**	-	-	+
**M 46**	4	2	**0.076**	**0.055**	+	-	-
**M 31**	8	13	3.273	3.086	+	+	+
**F 43**	7	3	3.257	3.422	-	+	-
**M 15**	7	12	3.497	3.311	-	+	-
**M 44**	5	17	3.23	3.284	-	+	-
**F 50**	7	2	**0.359**	**0.349**	-	+	-
**M 20**	10	2	3.586	3.308	-	+	+

**M 36**	7	2	1.505	2.838	-	+	-
**M 47**	1	14	**0.491**	**0.487**	-	+	-
**M 43**	5	6	**0.252**	2.485	+	+	-
**M 49**	8	15	1.904	2.299	-	+	+
**M 45**	5	16	1.889	2.121	+	+	-
**M 32**	7	13	**1.01**	2.605	-	+	+
**F 53**	7	21	2.083	2.008	-	+	+
**F 41**	4	13	2.794	3.401	-	+	-
**F 26**	10	14	3.339	3.545	-	+	+
**F 59**	2	1	**0.378**	**0.341**	-	-	+

NA = Not available

[a]Cut-off OD is 1.474. Bold fonts represent negative results

[b]A combination of PCR assays was defined as positive when at least two out of the three PCR assays (56kDa nested PCR (nPCR) assay, 47kDa-based quantitative real-time PCR (qPCR) assay, *GroEL*-based qPCR assay) were positive.
