# Supplementary material for: Optimal Cutoff and Accuracy of an IgM Enzyme-Linked Immunosorbent Assay for Diagnosis of Acute Scrub Typhus in Northern Thailand: an Alternative Reference Method to the IgM Immunofluorescence Assay
Source: J Clin Microbiol. 2016 May 23;54(6):1472–8. doi: 10.1128/JCM.02744-15 (PMC4879268; doi:10.1128/JCM.02744-15)
Supplement: Supplemental material [file JCM.02744-15_zjm999094981so3.pdf]

Figure S2. Unbiased receiver operating characteristic (ROC) curves using Bayesian latent class models (LCMs) for all possible cut-off OD of IgM ELISA at sample dilutions from 1:100 to 1:102,400 (A-K) using paired samples.

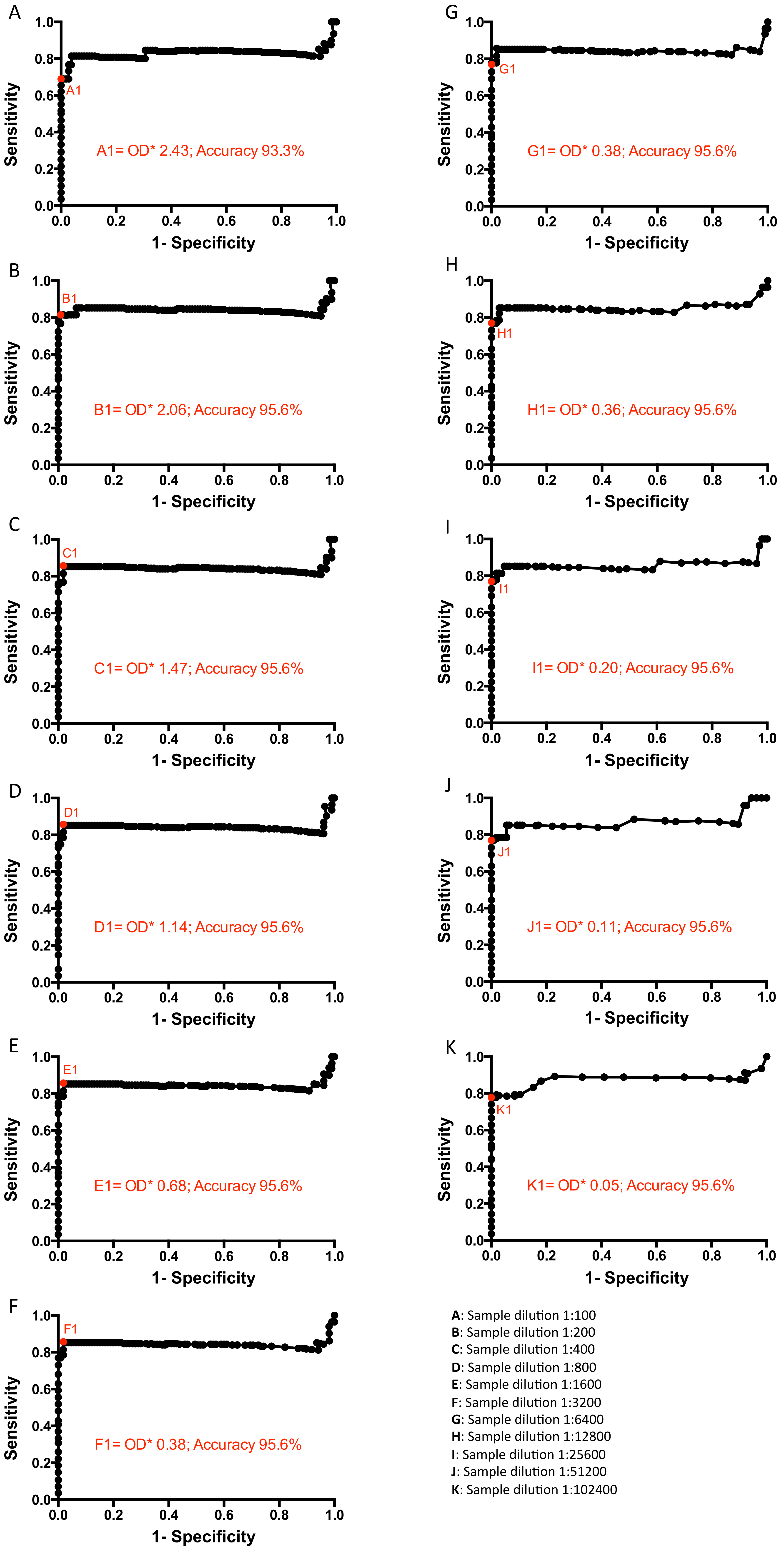

A: Sample dilution 1:100
B: Sample dilution 1:200
C: Sample dilution 1:400
D: Sample dilution 1:800
E: Sample dilution 1:1600
F: Sample dilution 1:3200
G: Sample dilution 1:6400
H: Sample dilution 1:12800
I: Sample dilution 1:25600
J: Sample dilution 1:51200
K: Sample dilution 1:102400
