# Supplementary material for: Optimal Cutoff and Accuracy of an IgM Enzyme-Linked Immunosorbent Assay for Diagnosis of Acute Scrub Typhus in Northern Thailand: an Alternative Reference Method to the IgM Immunofluorescence Assay
Source: J Clin Microbiol. 2016 May 23;54(6):1472–8. doi: 10.1128/JCM.02744-15 (PMC4879268; doi:10.1128/JCM.02744-15)
Supplement: Supplemental material [file JCM.02744-15_zjm999094981so4.pdf]

Supplementary Text 1. The data set for Bayesian latent class models.

The following is the complete data set for Bayesian latent class model to estimate the accuracies of diagnostic tests for scrub typhus with conditional dependence between IgM IFA and IgM ELISA. The first, second, third, fourth, and fifth columns are the results of *in vitro* isolation of *O. tsutsugamushi* (culture), a combination of PCR assays, admission IgM IFA (positive when IFA IgM in admission sample was ≥1:3,200), admission IgM ELISA (positive when Optical Density of IgM ELISA in admission sample at sample dilution 1:400 was ≥1.474) and presence of eschar, respectively. 1= positive result and 0= negative result. Each row represents one independent participant.

```
list( y=structure(
.Data=c(
       0,       0,       0,       0,       0,
       0,       1,       0,       0,       1,
       0,       0,       0,       0,       0,
       0,       1,       0,       0,       0,
       0,       0,       0,       0,       0,
       0,       0,       0,       0,       0,
       0,       0,       0,       0,       0,
       0,       1,       1,       0,       0,
       0,       0,       0,       0,       0,
       0,       0,       0,       0,       0,
       0,       0,       0,       0,       0,
       1,       0,       0,       0,       0,
       0,       0,       0,       0,       0,
       0,       0,       0,       0,       0,
       0,       0,       0,       0,       0,
       0,       0,       0,       0,       0,
       0,       0,       0,       0,       0,
       0,       0,       0,       0,       0,
       0,       0,       0,       0,       0,
       0,       0,       0,       0,       0,
       0,       0,       0,       0,       0,
       0,       0,       0,       0,       0,
       0,       0,       0,       0,       0,
       0,       0,       0,       0,       0,
       0,       0,       0,       0,       0,
       0,       0,       0,       0,       0,
       0,       0,       0,       0,       0,
       0,       0,       0,       0,       0,
       0,       0,       0,       0,       0,
       0,       0,       0,       0,       0,
       0,       0,       0,       0,       0,
       0,       0,       0,       0,       0,
       0,       0,       0,       0,       0,
       0,       0,       0,       0,       0,
       0,       0,       0,       0,       0,
       0,       0,       0,       0,       1,
       0,       0,       0,       0,       0,
       0,       0,       0,       0,       0,
       0,       0,       0,       0,       0,
       0,       0,       0,       0,       0,
       0,       0,       0,       0,       0,
       0,       0,       0,       0,       0,
       0,       0,       0,       0,       0,
       0,       0,       0,       0,       0,
       0,       0,       0,       0,       0,
       0,       0,       0,       0,       0,
       0,       0,       0,       0,       0,
       0,       0,       0,       0,       0,
       0,       0,       0,       0,       0,
       0,       0,       0,       0,       0,
       0,       0,       0,       0,       0,
       0,       0,       0,       0,       0,
       0,       0,       0,       0,       0,
       0,       0,       0,       0,       0,
```

```
0,        0,        0,        0,        0,
0,        0,        0,        0,        0,
0,        0,        0,        0,        0,
0,        0,        0,        0,        0,
0,        0,        0,        0,        1,
0,        0,        0,        0,        0,
0,        0,        0,        0,        0,
0,        0,        0,        0,        0,
0,        0,        0,        0,        0,
0,        0,        0,        0,        0,
0,        0,        0,        0,        0,
0,        0,        0,        0,        0,
0,        0,        0,        0,        0,
0,        0,        0,        0,        0,
0,        0,        0,        0,        0,
0,        0,        0,        0,        0,
0,        0,        0,        0,        0,
0,        0,        0,        0,        0,
0,        0,        0,        0,        0,
0,        0,        0,        0,        0,
0,        0,        0,        0,        0,
0,        0,        0,        0,        0,
0,        0,        0,        0,        0,
0,        0,        0,        0,        0,
0,        0,        0,        0,        0,
0,        0,        0,        0,        0,
0,        0,        0,        0,        0,
0,        0,        0,        0,        0,
0,        0,        0,        0,        0,
0,        0,        0,        0,        0,
0,        0,        0,        0,        0,
0,        0,        0,        0,        0,
0,        0,        0,        0,        0,
0,        0,        0,        0,        0,
0,        0,        0,        0,        0,
0,        0,        0,        0,        0,
0,        0,        0,        0,        0,
1,        0,        0,        0,        0,
0,        0,        0,        0,        0,
0,        0,        0,        0,        0,
0,        0,        0,        0,        0,
0,        0,        0,        0,        0,
0,        0,        0,        0,        0,
0,        0,        0,        0,        0,
0,        1,        1,        0,        0,
0,        0,        0,        0,        0,
0,        0,        0,        0,        0,
0,        0,        0,        0,        0,
0,        1,        0,        0,        0,
0,        0,        0,        0,        0,
0,        0,        0,        0,        0,
0,        0,        0,        0,        0,
0,        0,        0,        0,        0,
0,        0,        0,        0,        0,
0,        0,        0,        0,        0,
0,        0,        0,        0,        0,
0,        1,        0,        1,        0,
0,        1,        0,        0,        0,
0,        0,        0,        0,        0,
0,        0,        0,        0,        0,
0,        0,        1,        1,        1,
1,        1,        1,        1,        1,
0,        1,        1,        1,        1,
0,        0,        1,        1,        0,
0,        0,        1,        1,        1,
1,        1,        0,        0,        0,
1,        1,        1,        1,        0,
0,        0,        1,        1,        0,
0,        1,        1,        1,        1,
0,        0,        1,        1,        0,
0,        1,        1,        1,        0,
0,        1,        0,        0,        1,
0,        1,        1,        1,        0,
0,        0,        1,        1,        1,
1,        1,        1,        1,        1,
0,        1,        1,        1,        1,
1,        1,        1,        1,        1,
0,        1,        0,        1,        0,
0,        1,        1,        1,        0,
```

```
       0,       1,       1,       1,       0,
       0,       1,       1,       1,       1),.Dim=c(135,5))
pattern=structure(
.Data=c(
        1,      1,      1,      1,      1,
        1,      1,      1,      1,      0,
        1,      1,      1,      0,      1,
        1,      1,      0,      1,      1,
        1,      0,      1,      1,      1,
        0,      1,      1,      1,      1,
        1,      1,      1,      0,      0,
        1,      1,      0,      1,      0,
        1,      0,      1,      1,      0,
        0,      1,      1,      1,      0,
        1,      1,      0,      0,      1,
        1,      0,      1,      0,      1,
        0,      1,      1,      0,      1,
        1,      0,      0,      1,      1,
        0,      1,      0,      1,      1,
        0,      0,      1,      1,      1,
        1,      1,      0,      0,      0,
        1,      0,      1,      0,      0,
        1,      0,      0,      1,      0,
        1,      0,      0,      0,      1,
        0,      1,      1,      0,      0,
        0,      1,      0,      1,      0,
        0,      1,      0,      0,      1,
        0,      0,      1,      1,      0,
        0,      0,      1,      0,      1,
        0,      0,      0,      1,      1,
        1,      0,      0,      0,      0,
        0,      1,      0,      0,      0,
        0,      0,      1,      0,      0,
        0,      0,      0,      1,      0,
        0,      0,      0,      0,      1,
        0,      0,      0,      0,      0
),.Dim=c(32,5)))
```

The following is the complete data set for Bayesian latent class model to estimate the accuracies of diagnostic tests for scrub typhus with conditional dependence between IgM IFA and IgM ELISA. The first, second, third, fourth, and fifth columns are the results of *in vitro* isolation of *O. tsutsugamushi* (culture), a combination of PCR assays, paired IgM IFA (positive when IFA IgM in admission sample was ≥1:3,200 and/or 4-fold rise to ≥1:3,200 in convalescent sample compared to admission sample), IgM ELISA (positive when the Optical Density of IgM ELISA in admission sample or convalescent sample at sample dilution 1:400 was ≥1.474) and presence of eschar, respectively. 1= positive result and 0= negative result. Each row represents one independent participant.

```
list( y=structure(
.Data=c(
       1,       0,       0,       0,       0,
       0,       1,       1,       0,       1,
       0,       0,       0,       0,       0,
       0,       1,       0,       0,       0,
       0,       0,       0,       0,       0,
       0,       0,       0,       0,       0,
       0,       1,       1,       0,       0,
       0,       0,       0,       0,       0,
       0,       0,       0,       0,       1,
       0,       0,       0,       0,       0,
       0,       0,       0,       0,       0,
       0,       0,       0,       0,       0,
       0,       0,       0,       0,       0,
       0,       0,       0,       0,       0,
       0,       0,       0,       0,       0,
       0,       0,       0,       0,       0,
       0,       0,       0,       0,       0,
       0,       0,       0,       0,       0,
       1,       0,       0,       0,       0,
       0,       0,       0,       0,       0,
       0,       0,       0,       0,       0,
       0,       0,       0,       0,       0,
       0,       0,       0,       0,       0,
       0,       0,       0,       0,       0,
       0,       0,       0,       0,       0,
       0,       0,       0,       0,       0,
       0,       0,       0,       0,       0,
       0,       0,       0,       0,       0,
       0,       0,       0,       0,       0,
       0,       0,       0,       0,       0,
       0,       0,       0,       0,       0,
       0,       0,       0,       0,       0,
       0,       0,       0,       0,       0,
       0,       0,       0,       0,       0,
       0,       0,       0,       0,       0,
       0,       0,       0,       0,       0,
       0,       0,       0,       0,       0,
       0,       0,       0,       0,       0,
       0,       0,       0,       0,       0,
       0,       0,       0,       0,       0,
       0,       0,       0,       0,       0,
       0,       0,       0,       0,       0,
       0,       0,       0,       0,       0,
       0,       0,       0,       0,       0,
       0,       0,       0,       0,       0,
       0,       0,       0,       0,       0,
       0,       0,       0,       0,       0,
       0,       0,       0,       0,       0,
       0,       0,       0,       0,       0,
       0,       0,       0,       0,       0,
       0,       0,       0,       0,       0,
       0,       0,       0,       0,       0,
       0,       0,       0,       0,       0,
```

0,	0,	0,	0,	0,
0,	0,	0,	0,	0,
0,	0,	0,	0,	0,
0,	0,	0,	0,	0,
0,	0,	0,	0,	0,
0,	0,	0,	0,	0,
0,	0,	0,	0,	0,
0,	0,	0,	0,	0,
0,	0,	0,	0,	0,
0,	0,	0,	0,	0,
1,	1,	1,	0,	0,
0,	0,	0,	0,	0,
0,	0,	0,	0,	0,
0,	0,	0,	0,	0,
0,	0,	0,	0,	0,
0,	0,	0,	0,	0,
0,	0,	0,	0,	0,
0,	0,	0,	0,	0,
0,	0,	0,	0,	0,
0,	0,	1,	0,	0,
0,	0,	0,	0,	0,
0,	0,	0,	0,	1,
0,	0,	0,	0,	0,
0,	0,	0,	0,	0,
0,	0,	0,	0,	0,
0,	0,	0,	0,	0,
0,	0,	0,	0,	0,
0,	0,	0,	0,	0,
0,	0,	0,	0,	0,
0,	0,	0,	0,	0,
0,	0,	0,	0,	0,
0,	0,	0,	0,	0,
0,	0,	0,	0,	0,
0,	0,	0,	0,	0,
0,	0,	0,	0,	0,
0,	0,	0,	0,	0,
0,	0,	0,	0,	0,
0,	1,	0,	0,	0,
0,	0,	0,	0,	0,
0,	0,	0,	0,	0,
0,	0,	0,	0,	0,
0,	0,	0,	0,	0,
0,	0,	0,	0,	0,
0,	0,	0,	0,	0,
0,	0,	0,	0,	0,
0,	0,	0,	0,	0,
0,	1,	0,	0,	0,
0,	0,	0,	0,	0,
0,	0,	0,	0,	0,
0,	0,	0,	0,	0,
0,	0,	0,	0,	0,
0,	0,	0,	0,	0,
0,	0,	0,	0,	0,
0,	0,	0,	1,	0,
0,	0,	0,	1,	0,
0,	0,	0,	0,	0,
0,	0,	0,	0,	0,
0,	0,	0,	1,	0,
0,	1,	1,	1,	1,
0,	1,	1,	1,	0,
0,	0,	0,	0,	0,
0,	0,	0,	1,	0,
0,	1,	0,	1,	0,
0,	0,	1,	1,	0,
0,	1,	1,	1,	1,
0,	0,	1,	1,	1,
1,	1,	1,	1,	0,
0,	1,	1,	1,	0,
0,	0,	1,	1,	1,
0,	1,	1,	1,	1,
1,	1,	1,	1,	1,
0,	1,	1,	1,	0,
0,	1,	1,	1,	0,
0,	1,	1,	1,	1,
0,	0,	1,	1,	0,
0,	0,	1,	1,	1,
0,	0,	1,	1,	0,
1,	1,	1,	1,	1,
1,	1,	1,	1,	1,

```
       0,       1,       1,       1,       0,
       0,       1,       1,       1,       1,
       0,       1,       1,       1,       0),.Dim=c(135,5))
pattern=structure(
.Data=c(
       1,      1,      1,      1,      1,
       1,      1,      1,      1,      0,
       1,      1,      1,      0,      1,
       1,      1,      0,      1,      1,
       1,      0,      1,      1,      1,
       0,      1,      1,      1,      1,
       1,      1,      1,      0,      0,
       1,      1,      0,      1,      0,
       1,      0,      1,      1,      0,
       0,      1,      1,      1,      0,
       1,      1,      0,      0,      1,
       1,      0,      1,      0,      1,
       0,      1,      1,      0,      1,
       1,      0,      0,      1,      1,
       0,      1,      0,      1,      1,
       0,      0,      1,      1,      1,
       1,      1,      0,      0,      0,
       1,      0,      1,      0,      0,
       1,      0,      0,      1,      0,
       1,      0,      0,      0,      1,
       0,      1,      1,      0,      0,
       0,      1,      0,      1,      0,
       0,      1,      0,      0,      1,
       0,      0,      1,      1,      0,
       0,      0,      1,      0,      1,
       0,      0,      0,      1,      1,
       1,      0,      0,      0,      0,
       0,      1,      0,      0,      0,
       0,      0,      1,      0,      0,
       0,      0,      0,      1,      0,
       0,      0,      0,      0,      1,
       0,      0,      0,      0,      0
),.Dim=c(32,5)))
```
