# Supplementary material for: Optimal Cutoff and Accuracy of an IgM Enzyme-Linked Immunosorbent Assay for Diagnosis of Acute Scrub Typhus in Northern Thailand: an Alternative Reference Method to the IgM Immunofluorescence Assay
Source: J Clin Microbiol. 2016 May 23;54(6):1472–8. doi: 10.1128/JCM.02744-15 (PMC4879268; doi:10.1128/JCM.02744-15)
Supplement: Supplemental material [file JCM.02744-15_zjm999094981so5.pdf]

Supplementary Text 2. WinBUGS models.

The following is the WinBUGS codes for Bayesian latent-class model with conditional dependence between results of IgM IFA and IgM ELISA. The same model structure is applied to each and every dataset created

```
model{

for (i in 1:135){
    status[i]~dbern(prev)

###### Likellihood #######
for (j in 1:5){
    y[i,j]~dbern(p[i,j])
    ypred[i,j]~dbern(p[i,j])
    }
    }

for (i in 1:135){
for (j in 1:2){
    logit(p[i,j])<-status[i]*alpha[j]+(1-status[i])*beta[j]
}
for (j in 3:4){
    logit(p[i,j])<-status[i]*alpha[j]+(1-status[i])*beta[j]+status[i]*re[i]*IgM
    logit(pstatus[i,j])<-status[i]*alpha[j]+status[i]*re[i]*IgM+(1-status[i])*(-1000)
}
    logit(p[i,5])<-status[i]*alpha[5]+(1-status[i])*beta[5]
    re[i]~dnorm(0,1)
}

###### Prior ######
prev~dbeta(0.5,0.5)
IgM~dnorm(0.0,0.1)I(0,6)
alpha[1]~dnorm(0.0,0.1)
 logit(s[1])<-alpha[1]
beta[1]<--1000
for (j in 2:5){
       alpha[j]~dnorm(0.0,0.1)
       beta[j]~dnorm(0.0,0.1)I(,1)
       logit(s[j])<-alpha[j]
       logit(x[j])<--beta[j]
    }
se[1]<-s[1]
se[2]<-s[2]
se[5]<-s[5]
for (j in 3:4){
       se[j]<-sum(pstatus[,j])/sum(status[])
}

#### Unbiased estimation of sensitivity, specificity and accuracy #####
for (i in 1:135){
for (j in 1:5){
       diseasetestpos[i,j]<-status[i]*y[i,j]
       nondiseasetestneg[i,j]<-(1-status[i])*(1-y[i,j])
}
}
for (j in 1:5){
       sen[j]<-sum(diseasetestpos[,j])/sum(status[])
       spec[j]<-sum(nondiseasetestneg[,j])/(135-sum(status[]))
}

for (j in 1:5){
accuracy[j]<-(sum(diseasetestpos[,j])+ sum(nondiseasetestneg[,j]))/135
}

###### Prediction ######
for (i in 1:135){
for (k in 1:32){
for (j in 1:5){
    arraymatched[i,k,j]<-equals(ypred[i,j],pattern[k,j])
    }
    nmatched[i,k]<-sum(arraymatched[i,k,])
```

```
    matchedpattern[i,k]<-equals(nmatched[i,k],5)
    }
    }
for (k in 1:32){
    freqpred[k]<-sum(matchedpattern[,k])
}

####### Bayesian p value ########
for (i in 1:135){
for (k in 1:32){
for (j in 1:5){
    arraymatchedobs[i,k,j]<-equals(y[i,j],pattern[k,j])
    }
    nmatchedobs[i,k]<-sum(arraymatchedobs[i,k,])
    matchedpatternobs[i,k]<-equals(nmatchedobs[i,k],5)
    }
    }
for (k in 1:32){
    freqobs[k]<-sum(matchedpatternobs[,k])
}
for (k in 1:32){
pvalue[k]<-step(freqpred[k]-freqobs[k])
}
pvalue34<-step(sen[3]-sen[4]-0.005)+0.5*equals(sen[4],sen[3])
}
```
